# Supplementary material for: Evaluating the impact of curfews and other measures on SARS-CoV-2 transmission in French Guiana
Source: Nat Commun. 2021 Mar 12;12:1634. doi: 10.1038/s41467-021-21944-4 (PMC7955077; doi:10.1038/s41467-021-21944-4)
Supplement: Supplementary file 1 — Supplementary Information [file 41467_2021_21944_MOESM1_ESM.pdf]

Supplementary Information

Supplementary Methods

The main model we use here corresponds to the one described in [1] but without explicitly including the population age structure; it is schematically represented in the figure below.

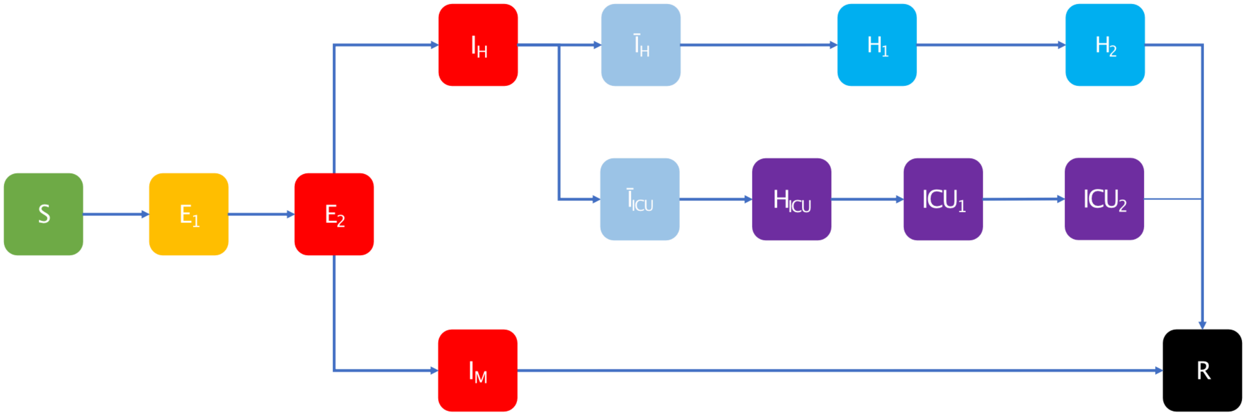

Upon infection, susceptible individuals (compartment S) become exposed while still not infectious (compartment E_1). Their subsequent entry in the E_2 compartment marks the start of the infectious period (compartments E_2 and I), which lasts until recovery (compartment R) or hospital admission. The I compartment is split into two compartments to represent individuals that develop mild (I_M) or severe disease (I_S). A subset of severely ill individuals further requires hospital admission (compartments $\bar{I}_H$, H_1, and H_2) or hospital and then ICU admission (compartments $\bar{I}_{ICU}$, H_{ICU}, ICU_1, and ICU_2).

The system of ordinary differential equations that governs the dynamics is then:

$$
\begin{aligned}
\frac{dS}{dt} &= -\beta S \frac{E_2 + I_M + I_H}{N} \\
\frac{dE_1}{dt} &= \beta S \frac{E_2 + I_M + I_H}{N} - \gamma_1 E_1 \\
\frac{dE_2}{dt} &= \gamma_1 E_1 - \gamma_2 E_2 \\
\frac{dI_M}{dt} &= (1 - p_H)\gamma_2 E_2 - \gamma_3 I_M \\
\frac{dI_H}{dt} &= p_H \gamma_2 E_2 - \gamma_3 I_H \\
\frac{d\bar{I}_H}{dt} &= (1 - p_{ICU})\gamma_3 I_H - \gamma_H^{in} \bar{I}_H \\
\frac{dH_1}{dt} &= \gamma_H^{in} \bar{I}_H - \gamma_H^{out} H_1 \\
\frac{dH_2}{dt} &= \gamma_H^{out} H_1 - \gamma_H^{out} H_2 \\
\frac{d\bar{I}_{ICU}}{dt} &= p_{ICU} \gamma_3 I_H - \gamma_{H,ICU}^{in} \bar{I}_{ICU} \\
\frac{dH_{ICU}}{dt} &= \gamma_{H,ICU}^{in} \bar{I}_{ICU} - \gamma_{ICU}^{in} H_{ICU} \\
\frac{dICU_1}{dt} &= \gamma_{ICU}^{in} H_{ICU} - \gamma_{ICU}^{out} ICU_1 \\
\frac{dICU_2}{dt} &= \gamma_{ICU}^{out} ICU_1 - \gamma_{ICU}^{out} ICU_2 \\
\frac{dR}{dt} &= \gamma_3 I_M + \gamma_H^{out} H_2 + \gamma_{ICU}^{out} ICU_2
\end{aligned}
\tag{1}
$$

where β is the transmission rate, $1/\gamma_1$ the time it takes for an exposed individual to become infectious (while still asymptomatic), $1/\gamma_2 + 1/\gamma_3 = D$ is the infectious period (so that $R_0 = \beta D$), p_H is the probability of hospitalization given infection, and p_{ICU} the probability of hospitalized individuals to require intensive cares. Following [1] we set: $1/\gamma_1$ = 4 days, $1/\gamma_2$ = 1 day, and $1/\gamma_3$ = 3 days, resulting in an incubation period of 5 days and in an infectious period of 4 days.

The other parameters represent the flow between compartments for severely ill individuals: γ_H^{in} (and $\gamma_{H,ICU}^{in}$) is the rate of hospital admissions for individuals not requiring intensive care (requiring intensive care), respectively, while γ_{ICU}^{in} is the rate of ICU admissions; finally, $2/\gamma_H^{out}$ is the time spent in the hospital general ward, and $2/\gamma_{ICU}^{out}$ is the time spent in ICU. Following [1] we set $1/\gamma_H^{in}$ = 4 days.

We fit our model to both admissions and number of occupied beds, so that the likelihood function is given by:

$$L = \prod_t dP(H_t|\overline{H}_t)\ dP(ICU_t|\overline{ICU}_t)\ dP(B_{H,t}|\overline{B}_{H,t})\ dP(B_{ICU,t}|\overline{B}_{ICU,t}) \tag{2}$$

where dP denotes the density of a Poisson distribution, t is the time point, H_t and ICU_t are the number of hospital and ICU admissions, $B_{H,t}$ and $B_{ICU,t}$ are the number of occupied general ward and ICU beds, and the barred variables correspond to expected values obtained by solving the system of differential equations above.

For one of our sensitivity analyses (see Supplementary Table 2 and Supplementary Figure 5), we included in the likelihood another Poisson term representing the contribution of the seroprevalence measured in [2]. More precisely, we added the term $dP(N_T|\overline{N}_T)$, with T = July 17 (the mid-point of the seroprevalence study period), N_T the estimated number of seropositive individuals at time T according to [2], and $\overline{N}_T$ the expected number of seropositive individuals at time T obtained from our model.

Supplementary Table 1. Model Parameters. [a] Value from [1], estimated using data from mainland France. [b] Values estimated using the mixture distribution described in [3] and using data from French Guiana available at the time the analyses were performed. All other values were estimated with our compartmental model.

**Parameter**	**Model M1**	**Model M2**	**Model M2 (Final)**
Initial number of infected individuals	413 [325, 514]	390 [310, 475]	365 [287, 453]
Days spent in hospital ($\tau_H = 2/\gamma_H^{out}$)	12.8[b]	11.8[b]	11.8[b]
Delay hospital to ICU admission in days ($\tau_{H,ICU} = 1/\gamma_{H,ICU}^{in}$)	1.5[a]	2.6[b]	2.6[b]
Days spent in ICU ($\tau_{ICU} = 2/\gamma_{ICU}^{out}$)	11.4[b]	17.6[b]	15.0 [13.1, 17.4]
Probability of ICU given hospitalization (p_{ICU})	21.5%[b]	11.0%[b]	15.7% [13.9%, 17.6%]
Reproduction number	1.35 [1.26, 1.45] (before 5/20)<br><br>1.78 [1.68, 1.88] (after 5/20)	1.40 [1.32, 1.49] (before 5/20)<br><br>1.71 [1.65, 1.77] (from 5/20 to 6/15)<br><br>1.14 [0.95, 1.31] (after 6/15)	1.44 [1.36, 1.53] (before 5/20)<br><br>1.69 [1.65, 1.73] (from 5/20 to 6/15)<br><br>1.08 [1.07, 1.10] (after 6/15)

Supplementary Figure 1. Sensitivity to severity and duration of stay in ICU. a Projected number of ICU beds according to different severity scenarios: baseline (p_H= 1.1%, red), low (p_H= 0.6%, green), and high (p_H= 1.8%, blue). **b** Projected number of ICU beds according to different durations of stay in ICU: baseline (τ_{ICU}= 11.4 days, red), short (τ_{ICU}= 8.0 days, brown), and long (τ_{ICU}= 15.0 days, purple). Solid lines indicate model posterior means while color areas indicate 95% credible intervals. Black dots indicate data used to calibrate the models, while empty circles denote data not available at the time of the analyses.

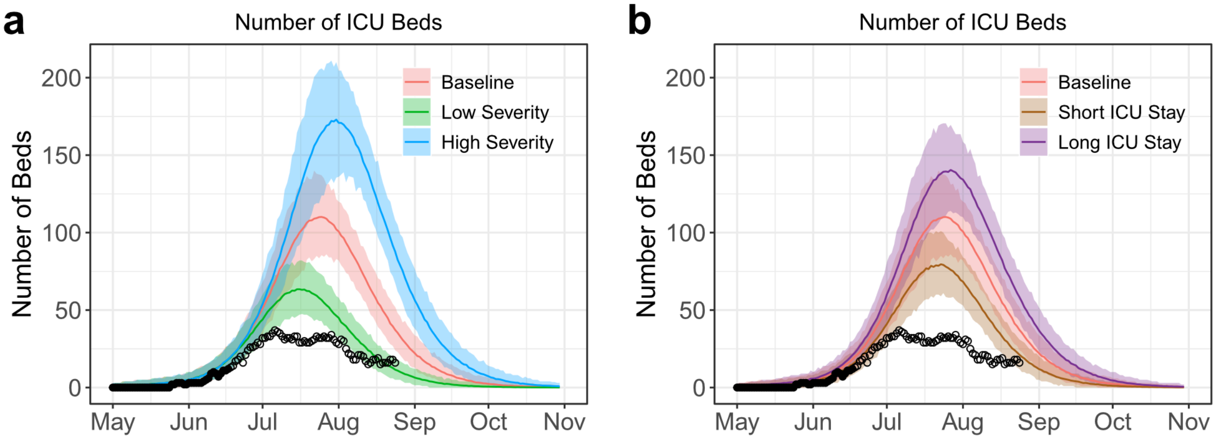

Supplementary Figure 2. Model selection. DIC difference (DIC of model M1 - DIC of model M2) for models calibrated from June 19th to June 29th. The dashed lines indicate a DIC difference of 4 units. Model M2 has a change point on June 15th (Supplementary Figure 3).

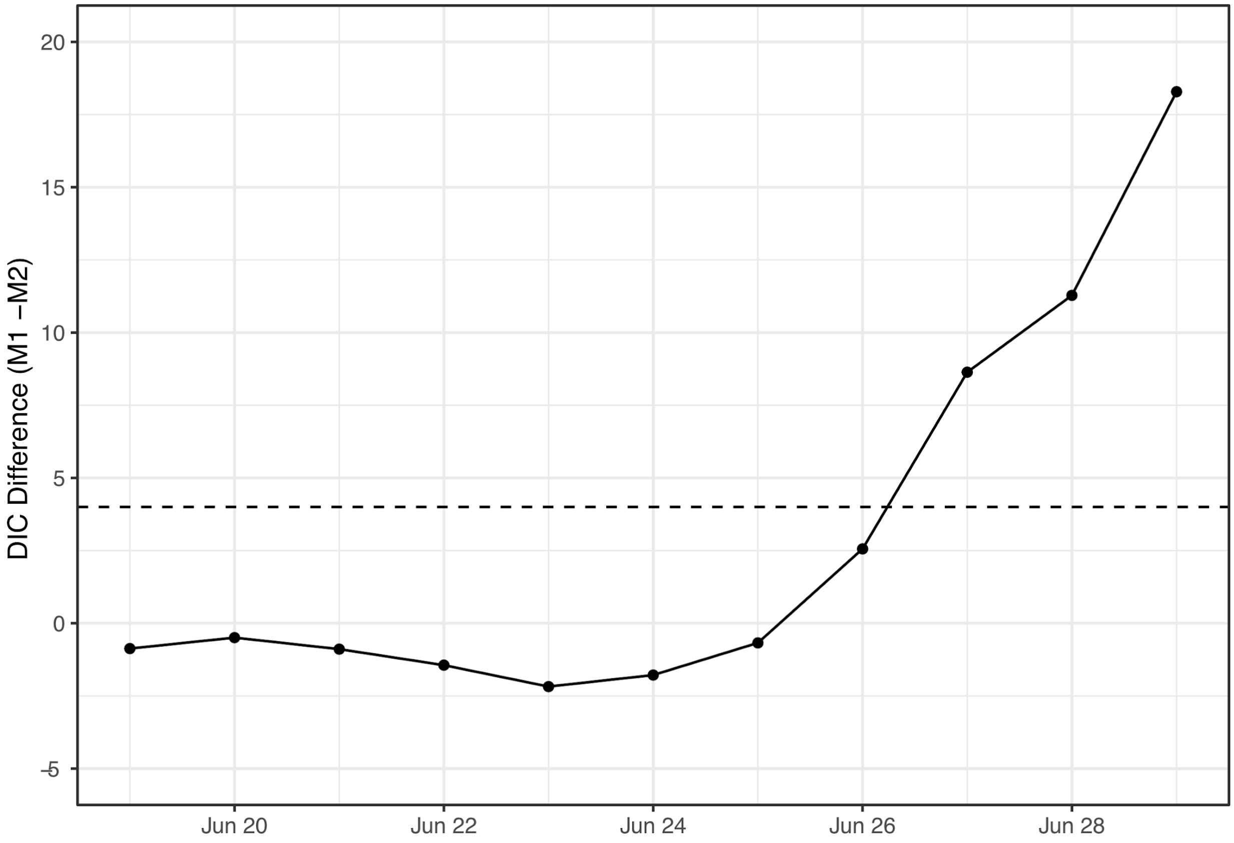

Supplementary Figure 3. Choice of transmission rate change point for model M2. Model DIC for change points ranging from June 6th to June 26th. The lowest DIC is obtained for a change point on June 15th. The dashed lines indicate a DIC difference of 4 units.

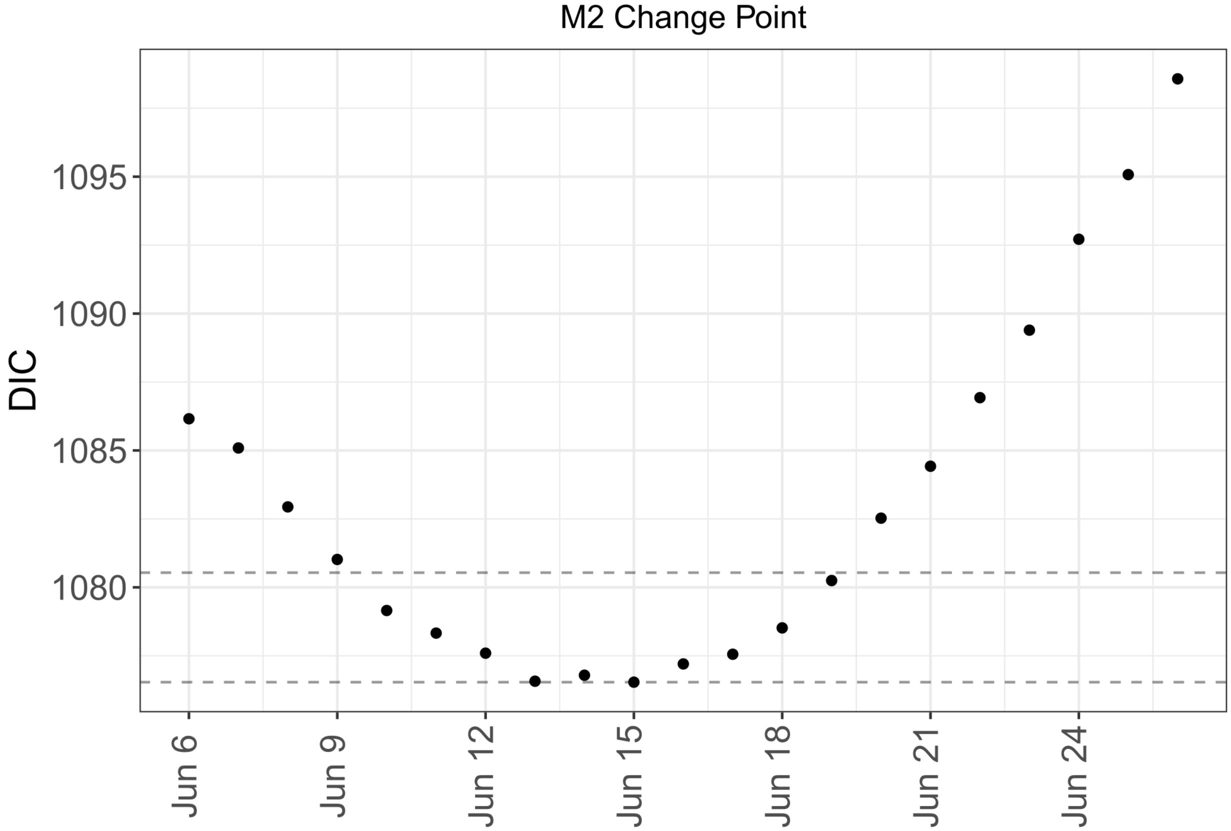

Supplementary Figure 4. Analyses made on August 25th 2020. Projections for the number of daily ICU (**a**) and hospital admissions (**b**), and ICU (**c**) and general ward (**d**) beds. Darker red colors correspond to older projections while lighter yellow colors correspond to more recent ones (from July 1st to August 26th). Black dots indicate actual data.

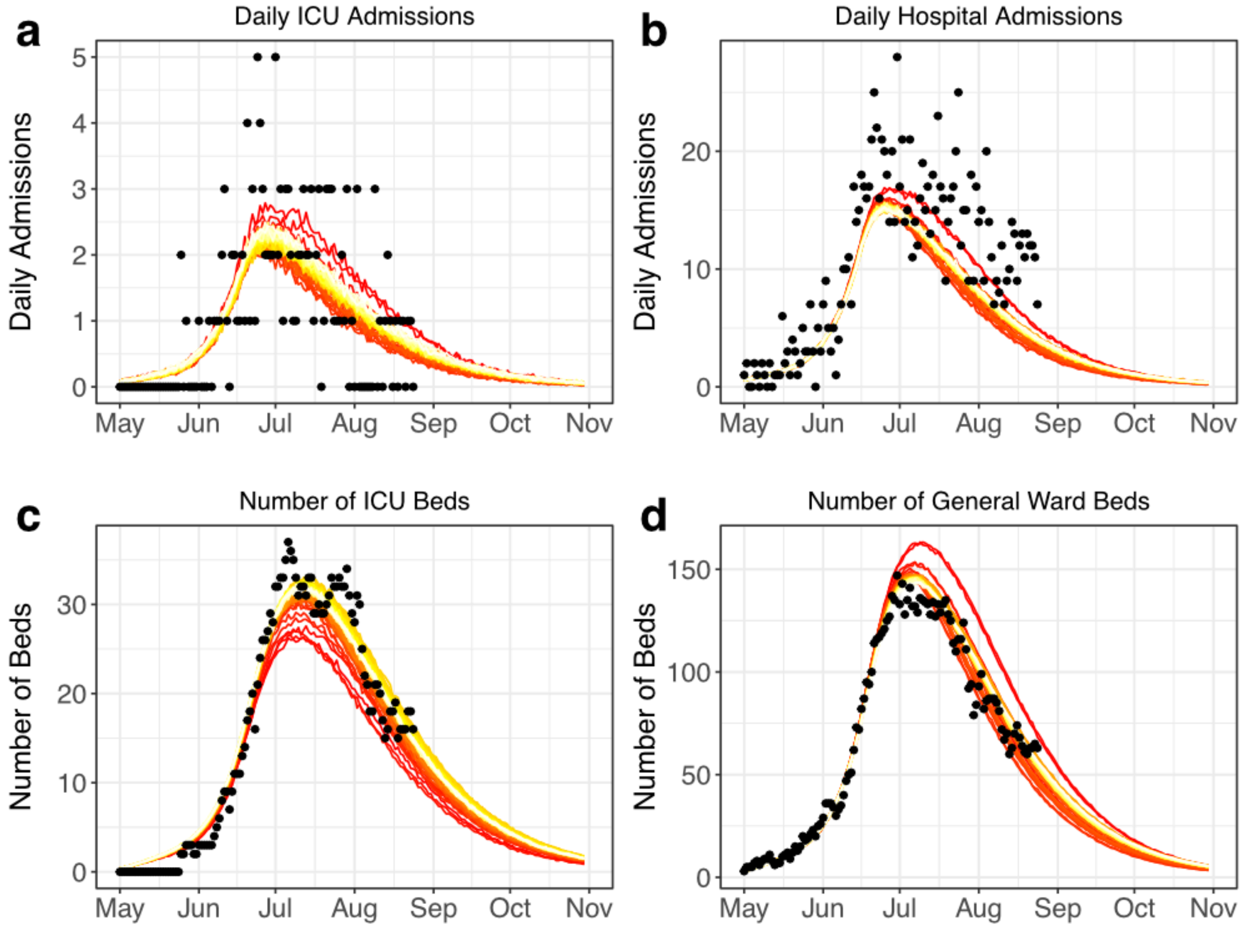

Supplementary Table 2. Adding seroprevalence estimates to the statistical framework. We used the seroprevalence estimates obtained by [2] and re-estimated our model free parameters. For comparison, the baseline model corresponds to the final model M2 in Supplementary Table 1. * Obtained using the estimates for metropolitan France discussed in [1] (see main text).

Parameter	Baseline Model	Modified Model
Probability of hospitalization upon infection	1.1%*	1.3% [1.2%, 1.3%]
Initial number of infected individuals	365 [287, 453]	322 [268, 380]
Days spent in ICU ($\tau_{ICU} = 2/\gamma_{ICU}^{out}$)	15.0 [13.1, 17.4]	15.1 [13.1, 17.2]
Probability of ICU given hospitalization (p_{ICU})	15.7% [13.9%, 17.6%]	15.7% [14.0%, 17.5%]
Reproduction number	1.44 [1.36, 1.53] (before 5/20)<br><br>1.69 [1.65, 1.73] (from 5/20 to 6/15)<br><br>1.08 [1.07, 1.10] (after 6/15)	1.44 [1.37, 1.51] (before 5/20)<br><br>1.68 [1.65, 1.72] (from 5/20 to 6/15)<br><br>1.05 [1.04, 1.06] (after 6/15)

Supplementary Figure 5. Per-Capita Hospitalizations in French Guiana and Metropolitan France. Number of hospital admission between March 1st and August 25th divided by the population size of each age group.

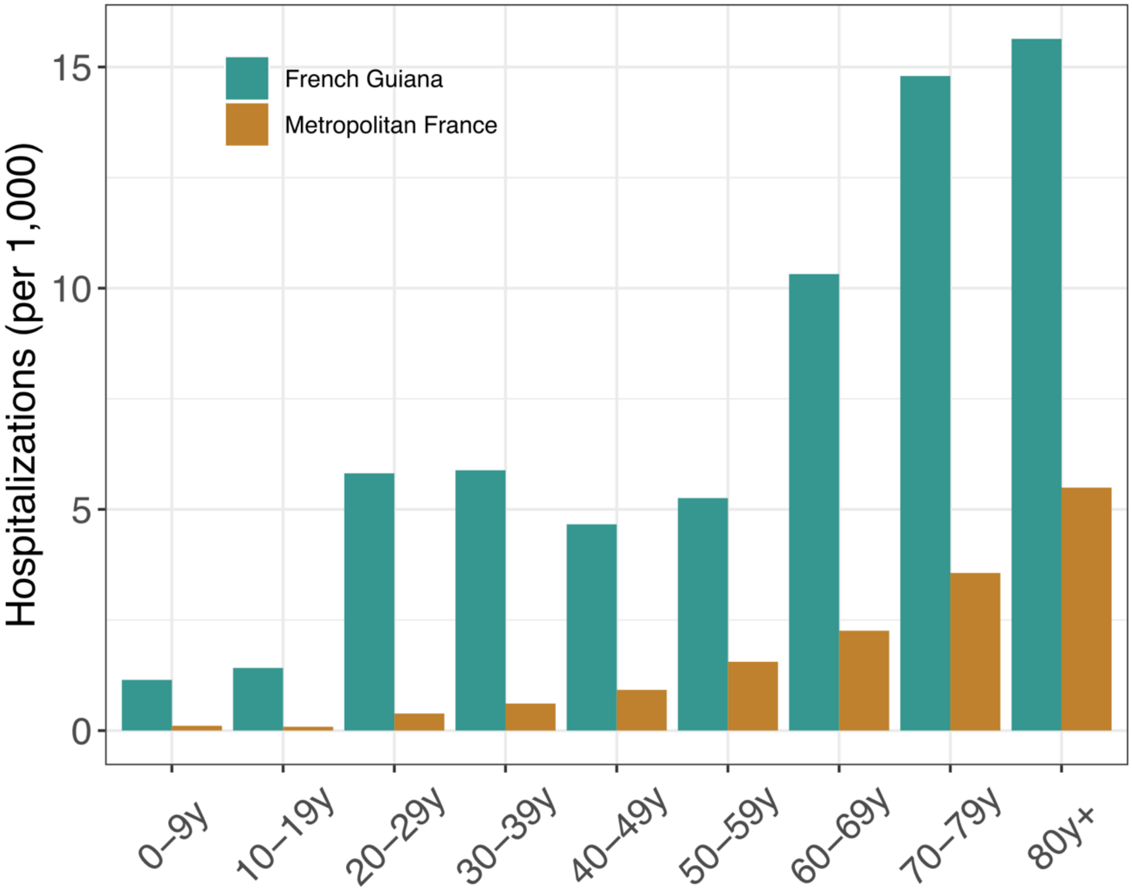

Supplementary Figure 6. Comparison between the final version of M2 and a modified version that includes seroprevalence estimates. We used the seroprevalence estimates obtained by [2]. Solid lines indicate model posterior means while color areas indicate 95% credible intervals. The baseline model - in blue - corresponds to the final model M2 in Supplementary Table 1.

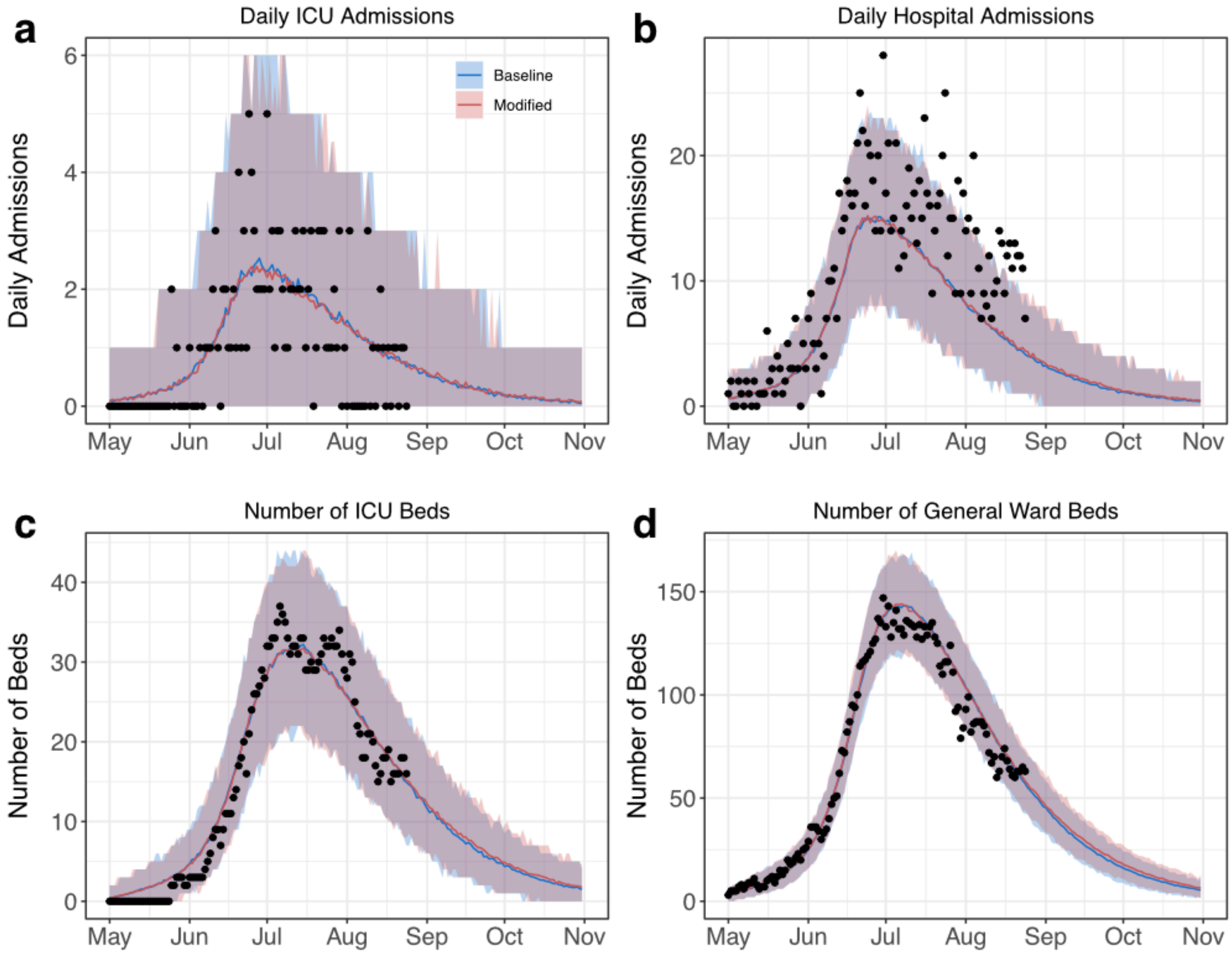

Supplementary Figure 7. Comparison between the final version of M2 and a version of M2 that includes age structure. Solid blue lines indicate model M2 posterior means while color areas indicate 95% credible intervals. The black lines denote trajectories obtained with the model that includes age structure: these were simulated by using the parameters' posterior means obtained by calibrating M2 to data available on August 25th.

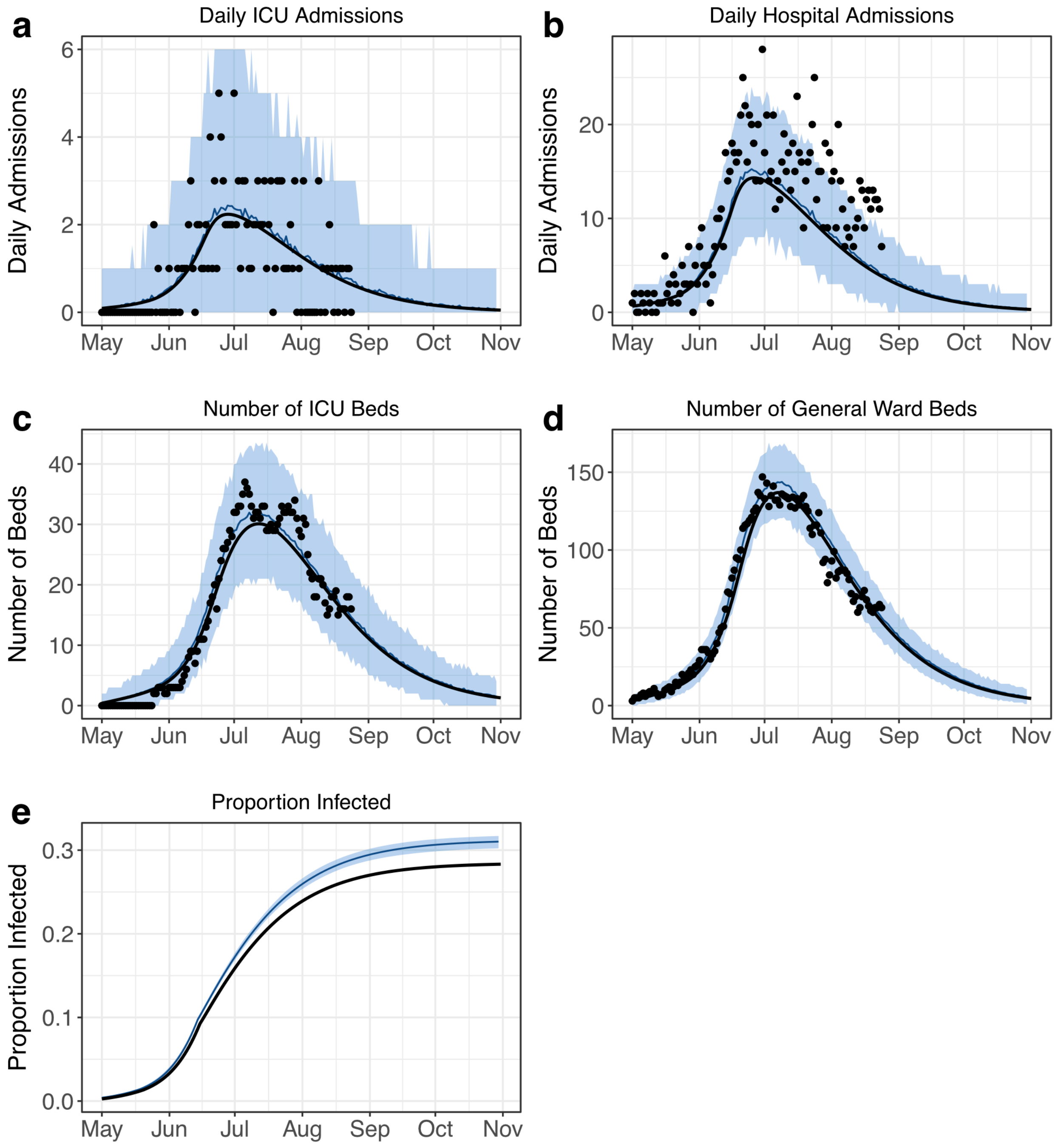

Supplementary References

1. Salje H, Tran Kiem C, Lefrancq N, Courtejoie N, Bosetti P, Paireau J, et al. Estimating the burden of SARS-CoV-2 in France. Science **369**, 208–211 (2020).

2. Flamand C, Enfissi A, Bailly S, Sarmento CA, Beillard E, Gaillet M, et al. Seroprevalence of anti-SARS-CoV-2 IgG at the epidemic peak in French Guiana. Preprint at https://www.medrxiv.org/content/10.1101/2020.09.27.20202465v1 (2020).

3. Lefrancq N, Paireau J, Hozé N, Courtejoie N, Yazdanpanah Y, Bouadma L, et al. Evolution of outcomes for patients hospitalized during the first SARS-CoV-2 pandemic wave in France. Preprint at https://hal.archives-ouvertes.fr/hal-02946545/document (2020).
